# Urinary Metabolic Profiles Before and After the Winter Training Season in Female Soccer Players

**DOI:** 10.3390/metabo16090675

**Published:** 2026-09-14

**Authors:** Hyang Yeon Kim, Jung Dae Lee, Ho-Seong Lee, Ji-Suk Chang, Suhkmann Kim, Gi-Wook Hwang, Kyu-Bong Kim

**Affiliations:** 1 College of Pharmacy, Dankook University, Cheonan 31116, Republic of Korea; festivalkim@gmail.com (H.Y.K.); ljd0734@nate.com (J.D.L.); 2 Center for Human Risk Assessment, Dankook University, 119 Dandae-ro, Cheonan 31116, Republic of Korea; 3 Department of International Sports Studies, Dankook University, Cheonan 31116, Republic of Korea; 4 Department of Sport Management, Dankook University, Cheonan 31116, Republic of Korea; longlivesports@dankook.ac.kr; 5 Department of Chemistry, Chemistry Institute for Functional Materials, Pusan National University, Busan Daehak-ro 63 beon-gil 2, Busan 46241, Republic of Korea; suhkmann@pusan.ac.kr; 6 Division of Environmental and Health Sciences, Faculty of Pharmaceutical Sciences, Toho ku Medical and Pharmaceutical University, 4-4-1 Komatsushima, Aoba-Ku, Sendai 981-8558, Miyagi, Japan; hwang@tohoku-mpu.ac.kr

**Keywords:** female athletes, winter training season (WTS), urinary metabolomics, NMR spectroscopy, recovery

## Abstract

**Background/Objectives**: Female soccer players differ from males in body composition, muscular strength, and hormonal fluctuations, which may influence performance, fatigue, recovery, and injury risk. This study aimed to characterize the time course of urinary metabolic perturbation and its persistence during a 20-day winter training season (WTS) using urinary metabolomics. **Methods**: Urinary metabolites in female soccer players were analyzed before (Bef) and 1 (After_1D) and 7 days after completion (After_7D) of the 20-day WTS using nuclear magnetic resonance (NMR) spectroscopy combined with multivariate analysis. **Results**: A total of 84 metabolites were identified in urine samples, and distinct group separation was observed by partial least squares discriminant analysis (PLS-DA) in targeted profiling. Among these, 13 metabolites, including adenine, alanine, citrate, creatine, creatine phosphate (PCr), formate, glutamine, glycine, malonate, mannitol, taurine, trimethylamine N-oxide (TMAO), and urea, showed significant changes in the three groups (Bef, After_1D and After_7D). **Conclusions**: Of the identified metabolites, six metabolites—alanine, PCr, formate, glutamine, glycine, and malonate—were significantly increased at 1 day post-WTS (After_1D) compared with pre-WTS (Bef) levels. These metabolites were lower at 7 days post-WTS (After_7D) than at 1 day post-WTS (After_1D). However, the metabolic profile had not fully returned to pre-WTS levels by day 7, suggesting that recovery following WTS may require longer than at least 7 days after a 20-day WTS.

## 1. Introduction

Female soccer is experiencing rapid growth worldwide, steadily strengthening its status as an international sport. According to Fédération Internationale de Football Association (FIFA), the number of registered female players and viewership of international competitions are steadily increasing, indicating that female soccer is moving beyond being a mere supplementary sport to becoming an independent competitive sport [1]. In particular, the World Cup and the Olympics are fueling growing social interest and academic research on female soccer.

Female and male soccer players share the same fundamental principles; however, female soccer players require more precise management of training load and recovery [2]. Female players differ from males in body composition, muscular strength, and hormonal fluctuations, which can influence performance, fatigue, recovery, and injury risk [3]. In addition, female players exhibit distinct physical, physiological, and match characteristics [4]. Compared to male soccer players, female soccer players tend to experience greater second-half fatigue [5]. Match profiles also differ, with male games typically characterized by higher overall intensity, while female games show differences in build-up play and passing speed [6]. These differences are also reflected in injury patterns. Male players more frequently experience posterior thigh injuries, whereas female players are more prone to ankle and knee injuries, including a higher risk of anterior cruciate ligament (ACL) injury [7]. To manage these demands, training load in female players is commonly monitored using indicators such as GPS, heart rate, and rating of perceived exertion, although the evidence base remains relatively limited compared to male soccer players [8]. Given the high physical demands of matches—including frequent high-intensity runs, accelerations, decelerations, and directional changes—post-match recovery is particularly important. Managing fatigue, muscle soreness, and sleep are therefore critical [8]. Accordingly, training for female players should be individualized, with greater emphasis on players’ responses to load rather than simply increasing training volume.

Winter training season (WTS) is essential to counteract off-season detraining and restore and improve aerobic capacity, strength, and power. It allows players to adapt physiologically to the high running demands and repeated high-intensity efforts required during the competitive season [9]. This period is also crucial for injury prevention, especially for lower-limb and ACL injuries, by developing neuromuscular control, strength, and change-of-direction ability [10]. Adequate pre-season conditioning creates a physical base that makes in-season load more manageable and helps maintain performance with lower injury risk [11]. Therefore, structured winter training is necessary not only for physical preparation, but also for optimizing tactical understanding, decision-making, and overall team readiness. For this reason, WTS is considered an essential training component for female soccer players. However, there is limited research on the recovery period following WTS. Although Kim et al. [12] suggested a recovery period for youth soccer players, differences in sex and age make direct comparisons difficult. Therefore, further research is needed to determine the recovery period following WTS in female soccer players.

In this context, metabolomics has emerged as a powerful tool for comprehensively characterizing metabolic responses to exercise and competition [13]. By simultaneously analyzing a wide range of low-molecular-weight metabolites, metabolomic approaches provide insights into energy metabolism, amino acid turnover, oxidative stress, and inflammatory responses associated with physical exertion [14]. In soccer research, metabolomics has been increasingly applied to evaluate exercise-induced metabolic perturbations, training adaptations, and recovery processes, offering a more integrated understanding of physiological demands beyond conventional performance or biochemical markers [15]. Recent studies have begun to apply metabolomic techniques to female athletes, including female soccer players, to investigate match-related metabolic changes and sex-specific metabolic responses to high-intensity intermittent exercise [3,14]. These studies suggest that female soccer matches induce significant alterations in metabolites related to glycolysis, lipid metabolism, and amino acid pathways, reflecting the unique energetic and physiological demands placed on female players. Moreover, metabolomic profiling has shown potential for identifying biomarkers associated with fatigue, muscle damage, and recovery status, which may be particularly relevant for optimizing training load and preventing injury in female soccer players.

Therefore, the present study aims to investigate metabolic responses in female soccer players during the WTS using a metabolomics-based approach, with the goal of providing scientific evidence to support optimized training prescription, recovery management, and the sustainable development of female soccer players.

## 2. Materials and Methods

### 2.1. Subjects

Sixteen female semi-professional soccer players were included in the final analysis. Of the 17 participants initially recruited, one participant withdrew before the 1-day post-WTS completion assessment and was therefore excluded from the 1-day and 7-day post-WTS completion analyses (Table 1). All participants were within the age range of 21 to 29 years old, were registered with the Korea Women’s Football Federation (KWFF) and competed in the WK (Women’s Korea) League. Weight, height, muscle mass, body fat and body mass index (BMI) were measured using the body composition analyzer (Inbody 770, Biospace, Seoul, Republic of Korea). The participants had a mean height of 165.5 ± 7.32 cm and a mean body weight of 59.6 ± 8.06 kg. The mean muscle mass was 25.3 ± 3.06 kg, while the mean body fat percentage was 14.0 ± 3.88%. The mean body mass index (BMI) was 21.7 kg/m^2^. All participants had more than one year of soccer experience. Also, they had been participating in the club for more than 2 h a day and 5 times a week or more. This study was approved by the Ethics Committee of Dankook University in accordance with the ethical standards of the Declaration of Helsinki (DKU 2020-07-004-001).

### 2.2. Winter Training Season (WTS) and Urine Collection

In the WTS program, the participants performed morning training for 150 min and afternoon training for 80 min, 5 days per week for 20 days (Table 2). The morning session consisted of a 10 min warm-up, 90 min of soccer training, 40 min of tactical training, and a 10 min cool-down. The afternoon session consisted of a 10 min warm-up, 60 min of circuit training, and a 10 min cool-down. Circuit training consisted of three sets of 20 repetitions at 40% of the one-repetition maximum (1RM). The exercise protocol followed established resistance training recommendations and a comparable circuit-based protocol previously used in soccer players [16,17,18]. Urine samples were collected at baseline before the 20-day WTS (Bef), as well as 1 day (After_1D) and 7 days after completion of the WTS (After_7D). At each time point, urine samples were collected before 10:00 a.m. in a fasting state and immediately stored at −70 °C until analysis.

### 2.3. ^1^H NMR Spectroscopic Analysis

Urine samples were processed for ^1^H NMR-based metabolomic analysis according to a previously established protocol with minor modifications [12]. After thawing at 4 °C, samples were centrifuged at 900× *g* for 5 min to remove insoluble materials. A total of 70 μL of D_2_O (Cambridge Isotope Laboratories, Tewksbury, MA, USA) containing 5 mM 4,4-dimethyl-4-silapentane-1-sulfonic acid (DSS; Sigma-Aldrich, St. Louis, MO, USA) and 100 mM imidazole (TCI Chemicals, Tokyo, Japan) was then mixed with 600 μL of the urine supernatant. To inhibit microbial contamination during sample acquisition, 30 μL of 0.42% sodium azide (Bio Basic Inc., Markham, ON, Canada) solution was additionally introduced. Prior to spectral acquisition, the pH of all samples was adjusted to 6.8 ± 0.2 using 1 M NaOH or 1 M HCl, as measured with a pH meter (METTLER TOLEDO, Gaithersburg, MD, USA). NMR spectra were acquired within 48 h using a Varian Unity Inova 600 MHz spectrometer (Varian, Inc., Palo Alto, CA, USA) at Pusan National University. Spectral acquisition was performed using a spectral width of 24,038.5 Hz, an acquisition time of 12.53 min, 128 scans, a relaxation delay of 3 s, and a saturation power setting of 4. Raw spectra were processed using VnmrJ 4.2 software (Agilent Technologies, Santa Clara, CA, USA), including phase adjustment, baseline correction, and chemical shift calibration relative to DSS at 0 ppm. The water resonance region (δ 4.5–5.0) was excluded prior to analysis to reduce interference from residual water signals. Metabolite assignment and quantification were conducted using Chenomx NMR Suite version 4.6 (Chenomx Inc., Edmonton, AB, Canada) in conjunction with the Chenomx reference library. Relative metabolite levels were normalized to creatinine to account for variation in urine concentration among samples.

### 2.4. Multivariate Analysis

Multivariate analysis was performed to assess overall metabolic variation and discrimination among the three sampling time points. Following quantification of metabolites in each spectrum in μM, the data were exported to Microsoft Excel (Microsoft Corp., Redmond, WA, USA) and subsequently imported into SIMCA-P software (version 12.0; Umetrics Inc., Kinnelon, NJ, USA) for multivariate statistical analysis. The data were Pareto-scaled prior to analysis. Principal component analysis (PCA) was first conducted as an unsupervised exploratory method to visualize the overall distribution, clustering patterns, and intrinsic variation in urinary metabolite profiles across baseline (Bef), 1 day after completion of the WTS (After_1D), and 7 days after completion of the WTS (After_7D). Partial least squares-discriminant analysis (PLS-DA) was subsequently performed as a supervised multivariate method to evaluate the overall separation among the three sampling time points. Orthogonal partial least squares-discriminant analysis (OPLS-DA) was subsequently performed for pairwise comparisons to maximize discrimination between groups and to identify metabolites contributing to group separation. Pairwise OPLS-DA models were constructed for Bef versus After_1D, Bef versus After_7D, and After_1D versus After_7D. Variable importance in projection (VIP) scores were calculated from each OPLS-DA model to evaluate the contribution of individual metabolites to group discrimination. Metabolites with VIP scores greater than 0.8 were selected as candidate discriminating metabolites and were subsequently evaluated using univariate statistical analysis.

### 2.5. Statistical Analysis

Univariate statistical analyses were subsequently performed to identify individual variables that differed among the sampling time points. Statistical analyses were performed using Excel 2013 (Microsoft, Windows version) and GraphPad Prism version 5.04 (GraphPad Software, San Diego, CA, USA). Differences in metabolite levels were analyzed using GraphPad Prism, and a *p*-value < 0.05 was considered statistically significant. Temporal changes in urinary metabolites following WTS were evaluated using a one-way repeated-measures ANOVA, followed by Tukey’s multiple comparison test when a significant effect of time was observed, to identify time points that differed significantly from baseline values.

## 3. Results

**Figure 1:**
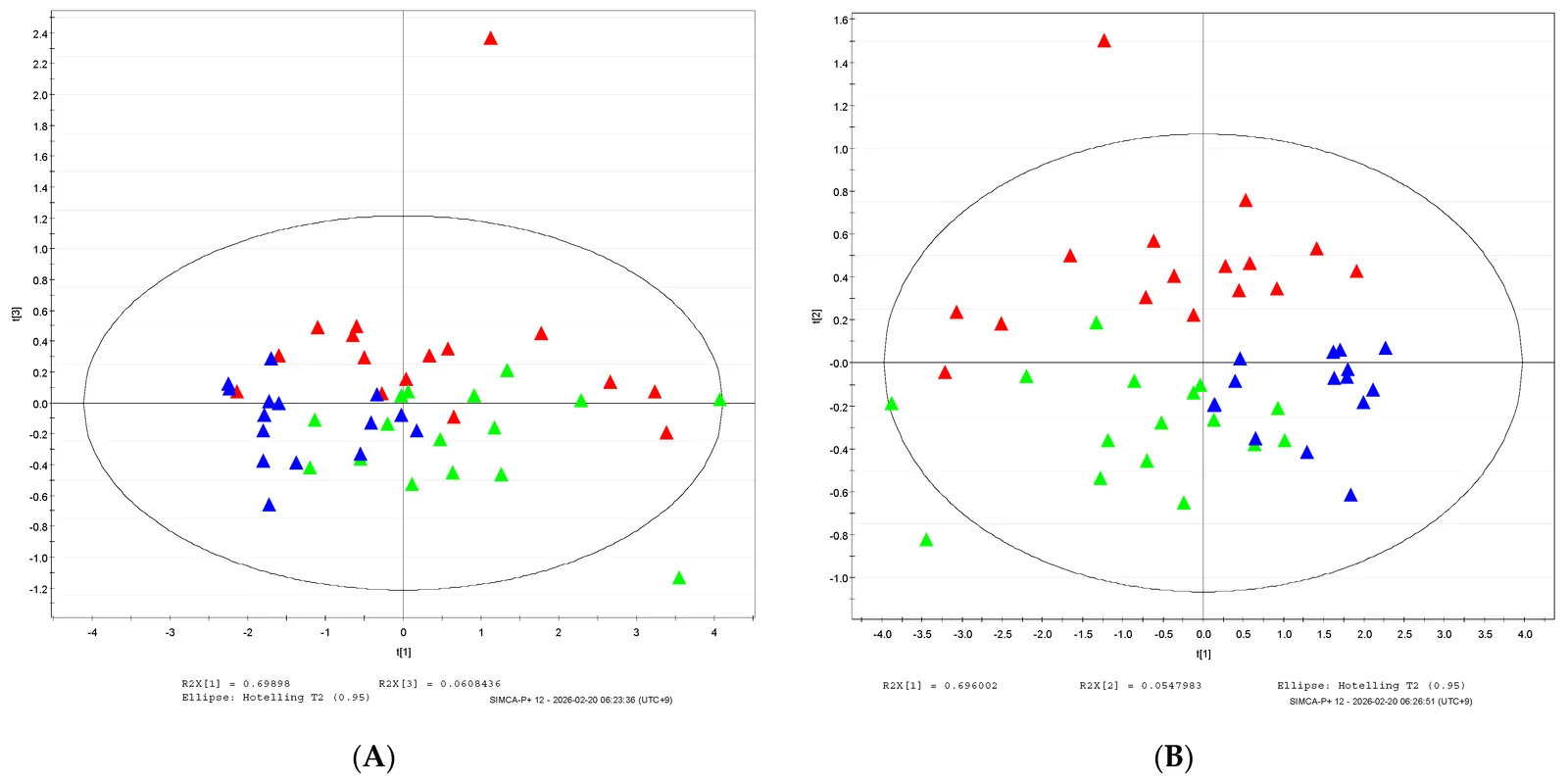
Multivariate analysis of urinary metabolite profiles at baseline (Bef, ▲), 1 day after completion of the winter training session (After_1D, ▲), and 7 days after completion of the winter training session (After_7D, ▲). (**A**) Principal component analysis (PCA) score plot (PC1, R^2^X = 0.699; PC3, R^2^X = 0.0608). (**B**) Partial least squares-discriminant analysis (PLS-DA) score plot (PLS1, R^2^X = 0.696; PLS2, R^2^X = 0.0548; R^2^Y(cum) = 0.484; Q^2^(cum) = 0.415).

For the urine samples obtained from female soccer players, 84 metabolites were assigned using the Chenomx NMR Suite program (Table 3). The PCA and PLS-DA score plots based on target profiling analysis showed partial and overlapping separation among the samples collected before the WTS (Bef) and at 1 day (After_1D) and 7 days (After_7D) after completion of the WTS (Figure 1).

**Figure 2:**
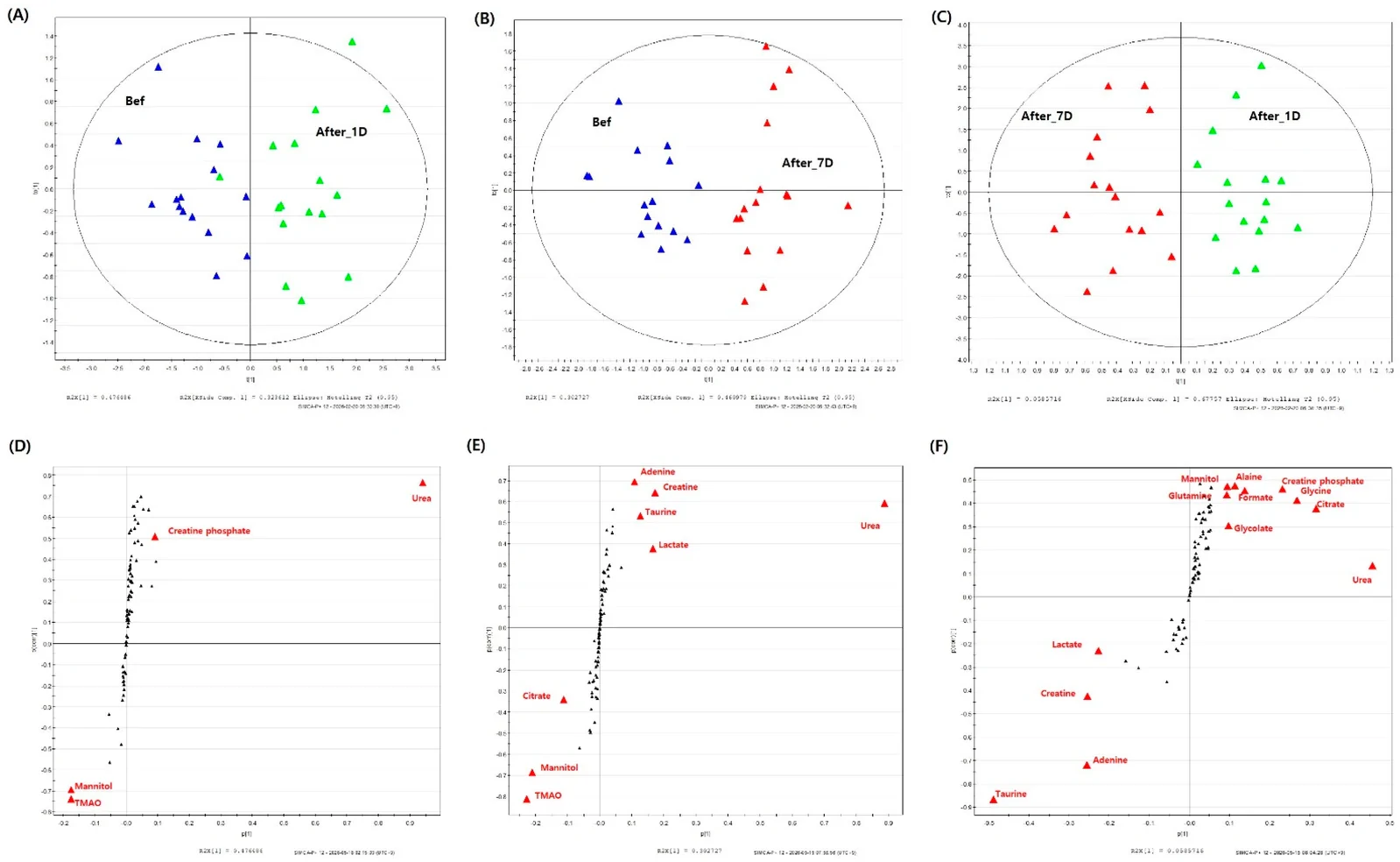
Orthogonal partial least squares-discriminant analysis (OPLS-DA) score plots (**A**–**C**) and corresponding S-plots (**D**–**F**) of identified urinary metabolites. (**A**,**D**) Baseline before the winter training session (Bef, ▲) versus 1 day after completion of the winter training session (After_1D, ▲) (R^2^X = 0.800, R^2^Y = 0.716, and Q^2^ = 0.576). (**B**,**E**) Bef versus 7 days after completion of the winter training session (After_7D, ▲) (R^2^X = 0.764, R^2^Y = 0.819, and Q^2^ = 0.787). (**C**,**F**) After_1D versus After_7D (R^2^X = 0.794, R^2^Y = 0.837, and Q^2^ = 0.625). The symbol ▲ in panels D−F indicates metabolites with VIP <0.8. WTS, winter training session.

In the PCA plot, the separation between Bef and After_1D was primarily observed along PC1, whereas the After_7D samples showed greater dispersion and were differentiated from the Bef and After_1D groups mainly along PC3, suggesting partial metabolic recovery with interindividual variability after 7 days of WTS. To identify the metabolic differences underlying the observed separations, VIP values were calculated using OPLS-DA for each pairwise comparison (Bef vs. After_1D, Bef vs. After_7D, and After_1D vs. After_7D) (Figure 2A–C, respectively). In addition, S-plots derived from the OPLS-DA models (Figure 2D–F) were used to visualize metabolites contributing to group separation.

**Figure 3:**
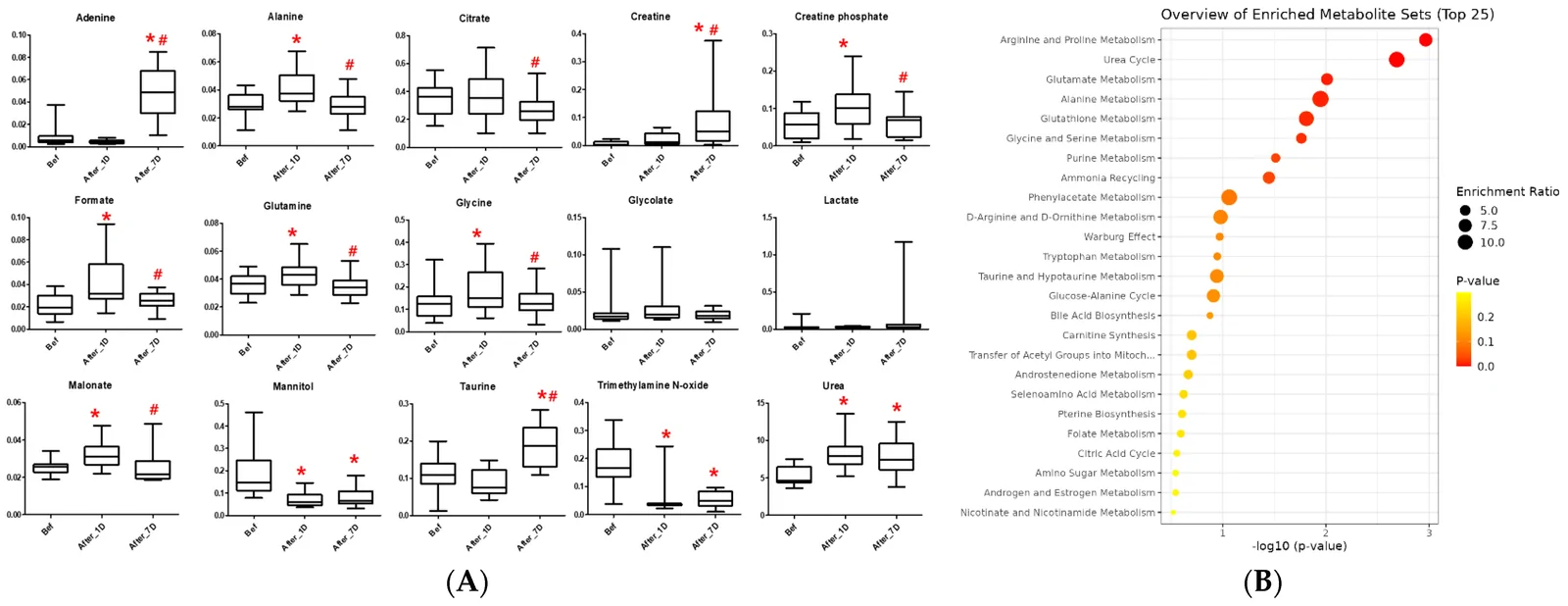
(**A**) Dot plots of selected urinary metabolites identified by orthogonal partial least squares-discriminant analysis (OPLS-DA). (**B**) Overview of the metabolic pathways associated with the selected metabolites. Data are presented as mean ± standard deviation. Bef, baseline before the winter training session; After_1D, 1 day after completion of the winter training session; After_7D, 7 days after completion of the winter training session. WTS, winter training session. *: *p* < 0.05 versus Bef, #: *p* < 0.05 versus After_1D.

Metabolites located at the extremities of the S-plots exhibited high covariance and high correlation and were considered key discriminatory variables. Based on VIP values (>0.8), 15 metabolites were selected and were highlighted as red dots in the S-plots. Of the 15 selected metabolites, 13 were identified as significantly different among the groups by ANOVA (*p* < 0.05), including adenine, alanine, citrate, creatine, creatine phosphate (PCr), formate, glutamine, glycine, malonate, mannitol, taurine, trimethylamine N-oxide (TMAO), and urea (Figure 3). Most metabolites exhibited a recovery trend toward baseline levels in the After_7D group, whereas adenine and creatine remained markedly altered. Among them, alanine, PCr, formate, glutamine, glycine, and malonate exhibited significant temporal changes, with their concentrations at 7 days after WTS (After_7D) approaching pre-WTS (Bef) levels.

## 4. Discussion

Targeted profiling of 84 urinary metabolites in female soccer players revealed partial discrimination among the three groups: before the WTS (Bef), 1 day after WTS (After_1D), and 7 days after WTS (After_7D). However, the greatest separation was observed between the Bef and After_1D groups by PC1 or PLS1 (Figure 1). These metabolic alterations were identified after completion of a 20-day WTS intervention involving around 4-h training sessions performed 5 days per week. Urinary urea, TMAO, mannitol and PCr were mainly affected by WTS (Figure 2A,D), and exercise-induced alterations in these metabolites have previously been reported in athletes, reflecting coordinated metabolic adaptations to physiological stress.

**Figure 4:**
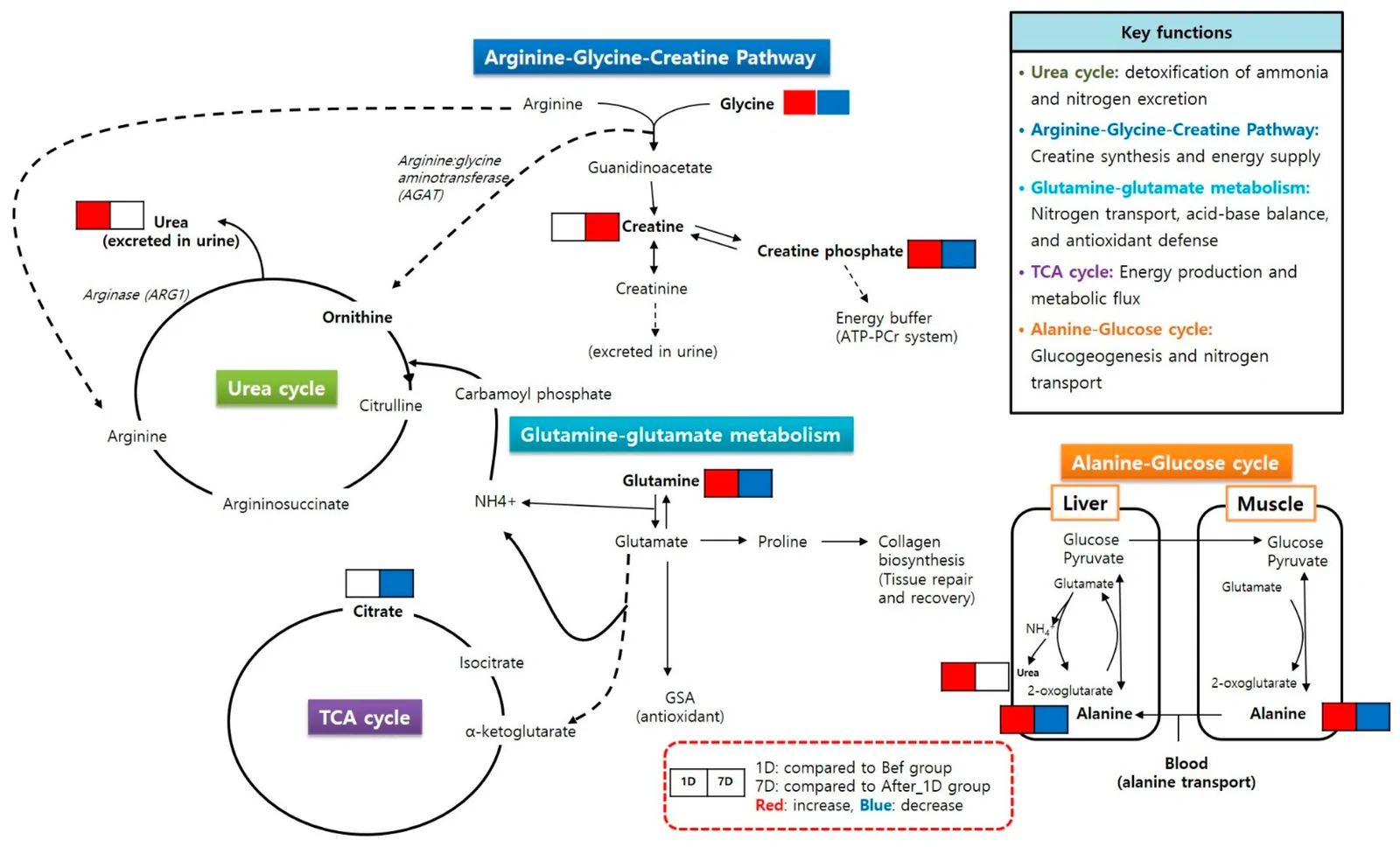
Metabolic pathway of major selected metabolites and their changes following the WTS. Bef: before WTS; After_1D: 1 day after WTS; After_7D: 7 days after WTS. In the figure, 1D indicates the comparison of After_1D with Bef, whereas 7D indicates the comparison of After_7D with After_1D. Red indicates an increase and blue indicates a decrease in urinary metabolite concentrations. The colored boxes represent changes in urinary concentrations and do not indicate measured tissue-level metabolic flux.

The elevated urea levels may be consistent with increased amino acid catabolism and nitrogen disposal via the urea cycle (Figure 4), potentially reflecting an increased reliance on protein metabolism when energy demand exceeds carbohydrate availability. In this study, some amino acids such as alanine, glutamine and glycine were one of the most highly increased in After_1D (Figure 3), suggesting increased protein breakdown and subsequent amino acid utilization for energy production and nitrogen disposal, as well as metabolic adaptation during recovery following intensive training. Of these metabolites, alanine is a key component of the glucose–alanine cycle (Figure 4), in which amino groups generated during exercise are transported from skeletal muscle to the liver [19,20]. In the liver, alanine serves as a substrate for gluconeogenesis, while its amino group is removed through the urea cycle. Therefore, the increase in alanine may reflect enhanced amino acid catabolism and gluconeogenic activity following exercise. Another amino acid, glutamine, serves as a key nitrogen carrier [21]. Through its conversion to glutamate and subsequently to α-ketoglutarate, glutamine can contribute to the replenishment of TCA cycle intermediates and energy production. Therefore, the increased glutamine level may reflect enhanced amino acid turnover and metabolic adaptation in response to exercise-induced energy demands. The last selected amino acid, glycine, contributes to activation of the arginine–glycine–creatine pathway (Figure 4) [22]. In the first step of this pathway, glycine combines with arginine to form guanidinoacetate, which is subsequently converted to creatine. As creatine and PCr play critical roles in rapid ATP regeneration during high-intensity exercise, the increased glycine level may reflect enhanced creatine turnover and metabolic adaptation to exercise-induced energy demands. In addition, during this process, the conversion of arginine and glycine to guanidinoacetate generates ornithine, a key intermediate of the urea cycle. Therefore, increased glycine metabolism may be associated with enhanced nitrogen disposal and ammonia detoxification following exercise. Collectively, these observations are compatible with the idea that amino acid metabolism was markedly altered following WTS, reflecting coordinated adaptations in energy production, nitrogen transport, creatine turnover, and recovery processes.

As mentioned above, exercise promotes amino acid catabolism, during which ammonia is produced as a major metabolic byproduct. Because ammonia is highly toxic, it must be detoxified and eliminated from the body via the urea cycle [23]. In this process, ammonia is first incorporated into carbamoyl phosphate and subsequently combines with ornithine to form citrulline, ultimately leading to the production of urea from arginine by arginase (ARG1), which is excreted by the kidneys. In parallel, arginine is also utilized in creatine biosynthesis, where it combines with glycine to form guanidinoacetate and ornithine via arginine:glycine amidinotransferase (AGAT). Previous research [24] has reported increased ARG1 expression in peripheral blood mononuclear cells 2 h after acute resistance exercise, suggesting that alterations in arginine metabolism may accompany the early tissue remodeling and repair response to exercise. In addition, exercise can induce transient muscle damage and inflammatory responses, followed by the activation of anti-inflammatory and tissue repair pathways. During tissue injury and repair, ARG1 expression may be upregulated in macrophages and has been associated with wound healing and extracellular matrix remodeling [25].

Moreover, exercise-induced hypoxia has been reported to downregulate AGAT expression in rats, potentially affecting creatine biosynthesis and PCr/creatine metabolism. In the present study, the observed increase in urinary PCr-related metabolites may be associated with alterations in creatine-related metabolism following intensive exercise. However, because PCr is predominantly an intracellular high-energy phosphate compound and the present study did not directly assess intramuscular PCr content or phosphagen turnover, urinary PCr should not be considered a direct indicator of muscle phosphagen-system activation. In muscle cells, creatine is reversibly phosphorylated to PCr by creatine kinase, providing a rapidly available energy buffer during muscle contraction [26]. Therefore, the observed urinary PCr changes may reflect systemic or renal handling of creatine-related metabolites rather than direct evidence of increased intramuscular phosphagen turnover.

Mannitol is primarily derived from dietary sources and is partially absorbed in the intestine, after which it is excreted largely unchanged in urine due to minimal metabolism and limited renal reabsorption [27]. Given its dietary origin, the observed decrease in urinary mannitol may primarily reflect differences in dietary intake between the pre-WTS and post-WTS collection periods. Exercise-related alterations in osmotic balance, hydration status, renal handling, or gastrointestinal permeability may also potentially influence urinary mannitol levels; however, these factors were not assessed in the present study. Although exercise-induced reductions in splanchnic blood flow have been suggested to affect intestinal permeability, the contribution of this mechanism to the observed change in urinary mannitol remains unclear [28].

In cells, trimethylamine N-oxide (TMAO), a gut microbiota-derived metabolite, is involved in maintaining cellular osmotic balance under conditions of physiological stress [29], and its decrease may reflect adaptive osmotic regulation and cellular stress responses induced by exercise [30]. Exercise has been shown to modulate gut microbiota composition and metabolite profiles [31]; however, evidence regarding exercise-induced changes in TMAO production remains inconsistent. In particular, acute intermittent exercise has been reported not to significantly alter TMAO production. Therefore, the decrease in urinary TMAO observed following WTS should not be interpreted as a direct exercise-induced change in TMAO metabolism, and potential contributions from exercise-related osmotic regulation or gut–liver metabolic alterations remain speculative.

In addition, urinary taurine has been reported to change following exhaustive exercise. Cuisinier et al. [32] reported that plasma taurine concentrations increased significantly immediately after a marathon and remained elevated 24 h later, whereas urinary taurine excretion tended to increase immediately after the race but did not remain elevated at 24 h. However, this study involved a single bout of prolonged marathon exercise and differed from the present 20-day intermittent team-sport training protocol in exercise modality, duration, participant characteristics, and sampling schedule. These findings therefore provide contextual evidence that exhaustive exercise can alter taurine metabolism but cannot be directly extrapolated to the present training protocol. A review of human studies [33] reported that taurine supplementation may attenuate exercise-induced muscle damage and soreness and improve some oxidative stress-related markers; however, these findings are not directly applicable to endogenous urinary taurine responses. Therefore, the decrease in urinary taurine observed in the present study should be interpreted cautiously, and its underlying mechanism remains unclear.

As mentioned above, amino acid catabolism during exercise may influence one-carbon metabolism, in which serine and glycine can contribute to formate production [34]. In the present study, urinary formate increased after exercise, which may be consistent with alterations in one-carbon metabolism; however, one-carbon metabolic flux and the activities of related enzymes were not directly measured. Malonate also showed a significant increase at After_1D, whereas citrate did not show a significant increase compared with Bef and instead showed a significant decrease from After_1D to After_7D. These findings may reflect transient alterations in mitochondrial and energy-related metabolism following exercise, although direct changes in tissue-level metabolic flux cannot be inferred from urinary metabolite concentrations alone. Citrate is associated with increased TCA cycle activity [35], while malonate and formate are associated with mitochondrial metabolism and the one-carbon metabolic pathway, respectively [36], but their urinary changes should be interpreted as changes in metabolite levels rather than direct evidence of altered pathway flux.

Although training load was comparable between the youth and female soccer players, this discrepancy may be attributed to differences in metabolic response and recovery kinetics, as alanine reflects both acute metabolic responses and post-exercise recovery processes [37]. Differences in training structure (morning training for 90 min and afternoon training for 60 min) [12], including the frequency of match play in the youth cohort, may have resulted in greater cumulative fatigue for youth soccer players, which could contribute to the delayed alanine peak. In addition, differences in muscle mass between groups may also partly contribute to the observed variation in alanine levels. As alanine is primarily synthesized and released from skeletal muscle, with its release increasing in response to exercise intensity and workload [38], lower muscle mass in younger athletes (15.8 ± 1.8 kg) compared with female soccer players (25.3 ± 3.06 kg) may influence the magnitude and timing of alanine release. In addition, age-related physiological differences may further affect these responses, as younger athletes are characterized by higher rates of protein turnover and ongoing growth-related metabolic activity, which can alter recovery kinetics and delay the peak of alanine following exercise [37,38]. However, these differences are more likely driven by variations in metabolic kinetics rather than muscle mass or age alone.

During prolonged and intensive exercise, increased production of reactive oxygen species (ROS) and activation of immune responses can contribute to exercise-induced muscle damage [39]. Consequently, exercise-induced metabolic alterations may serve as indicators of muscle damage and subsequent recovery processes. Jang et al. [40] investigated urinary metabolic changes following exercise-induced muscle damage (EIMD) in both male and female participants and identified adenine, glycine, creatine phosphate (PCr), and citrate as potential biomarkers. Notably, these metabolites were also identified in the present study. Therefore, the observed alterations in these metabolites may reflect muscle damage and associated metabolic adaptations induced by WTS. These data suggested that the observed metabolic alterations may reflect not only increased energy demands but also muscle repair and recovery processes after intensive training.

When urinary metabolites were monitored for up to 7 days after exercise, 6 out of the 15 metabolites (alanine, PCr, formate, glutamine, glycine, and malonate) showed significant recovery patterns by day 7 (Figure 3). However, adenine and creatine dramatically increased in the After_7D group compared to the Bef or After_1D group. This might suggest that the associated metabolic pathway remained active during the recovery period, rather than returning immediately to the pre-exercise state [41]. The progressive increase in urinary adenine during the recovery period may reflect sustained purine nucleotide turnover following exercise. As ATP degradation products continue to be recycled through the purine salvage pathway, elevated adenine excretion may indicate ongoing remodeling of nucleotide metabolism required for restoration of intracellular adenine nucleotide pools. In addition, creatine plays a central role in the creatine kinase/PCr system, which buffers cellular ATP availability during and after exercise [42]. The delayed increase in urinary creatine may reflect ongoing restoration of skeletal muscle creatine pools and continued metabolic remodeling associated with recovery. During the recovery period, creatine synthesis, transport, and utilization may become more active, resulting in increased creatine turnover and potentially greater urinary creatine excretion. Although urinary creatine does not directly reflect intramuscular creatine content, the sustained elevation observed in the present study suggests that creatine metabolism remains active during post-exercise recovery. Consistent with the physiological importance of creatine during recovery, previous studies have demonstrated that creatine supplementation increases intramuscular creatine availability, enhances phosphocreatine resynthesis, and may improve post-exercise recovery and repeated high-intensity exercise performance [43].

In this study, most of the selected metabolites that changed at 1 day post-WTS showed levels closer to pre-WTS values by 7 days post-WTS, indicating a substantial normalization of the urinary metabolomic profile during the post-WTS period. However, several metabolites remained different from their pre-WTS levels at 7 days, suggesting that metabolic normalization was not complete within this period. The selected metabolites were primarily related to energy metabolism, amino acid metabolism, osmotic regulation, and one-carbon metabolism, although the present urinary metabolomics data do not allow direct inference of tissue-level metabolic pathway activity or recovery. Taken together, these findings suggest that, from a urinary metabolomic perspective, metabolic recovery following WTS may extend beyond 7 days in female soccer players.

However, this study has several limitations. Objective internal and external training loads were not quantified, and physical activity, dietary intake, hydration status, and supplement use during the 7-day post-WTS period were not systematically monitored. In addition, menstrual-cycle phase and hormonal contraceptive use were not documented, which may be relevant to metabolic and urinary variations in this female athlete cohort. Recovery was not assessed using functional, performance-based, or independent biochemical outcomes; therefore, the observed movement of urinary metabolites toward pre-WTS levels should be interpreted as partial normalization of the urinary metabolomic profile rather than complete physiological recovery. Despite these limitations, the present study provides preliminary longitudinal data describing urinary metabolomic changes in female athletes before WTS and at 1 and 7 days after WTS. The repeated-measures design enabled the evaluation of within-participant temporal changes and reduced, to some extent, the effect of baseline interindividual metabolic heterogeneity. The observed post-WTS changes and subsequent trends toward pre-WTS levels identify candidate urinary metabolites that may be useful for future studies investigating training-related metabolic responses. Although these findings do not establish complete physiological recovery, they provide a basis for designing more comprehensive studies that combine metabolomics with objective training-load assessment, dietary and hydration monitoring, menstrual-cycle information, and functional recovery measures.

## 5. Conclusions

Urinary metabolite profiling in female soccer players before and after 1 and 7 days of winter training season (WTS) was performed using NMR spectroscopy combined with multivariate analysis to estimate an appropriate recovery period. A total of 84 metabolites were identified, and distinct group separation was observed in PLS-DA. Key discriminatory metabolites included adenine, alanine, citrate, creatine, creatine phosphate (PCr), formate, glutamine, glycine, malonate, mannitol, taurine, trimethylamine N-oxide (TMAO), and urea, which are associated with energy metabolism, amino acid catabolism, osmotic regulation, and one-carbon metabolism. Among these, six metabolites, alanine, PCr, formate, glutamine, glycine, and malonate, showed significant alterations at 1 day post-WTS and partially normalized the urinary metabolomic profile toward baseline levels by day 7. Collectively, these findings suggest that, from a urinary metabolomic perspective, metabolic recovery following WTS may extend beyond 7 days in female soccer players. Longer follow-up studies are warranted to determine the time required for complete metabolic normalization and to establish an appropriate recovery period.

## Figures and Tables

**Table 1:** Physical characteristics of the subjects.

N	Age (yrs)	Height (cm)	Weight (kg)	Muscle Mass (kg)	Body Fat (%)	BMI (kg/m^2^)
17	27.2 ± 4.32	165.5 ± 7.32	59.6 ± 8.06	25.3 ± 3.06	14.0 ± 3.88	21.7 ± 3.52

Data are reported as means ± standard deviation.

**Table 2:** Winter training program.

Session	Order	Type	Time	Intensity
Morning session	Warm-up	Stretching, running	10 min	
	Soccer training	Coordination	15 min	Rest between training: 5 min
		5:2 passing	15 min	
		Passing training	30 min	
		Passing game	30 min	
	Tactical training	Strategies set piece	40 min	
	Cool-down	Stretching	10 min	
Afternoon session	Warm-up	Stretching	10 min	
	Circuit training	Leg extension, leg curls, hip adduction, hip abduction, Smith squats, pushdowns, seated rows, shoulder press, chest press, chin-ups, chest fly, arm curls, push-ups, sit-ups	60 min	40% 1 RM 20 reps/3 sets rest between sets: 5 min
	Cool-down	Stretching	10 min	

**Table 3:** Fold changes in urinary metabolites identified by NMR-based targeted profiling following WTS.

No.	Metabolites	After_1D/Bef	After_7D/Bef	After_7D/After_1D
1	1,3-Dihydroxyacetone	1.28	1.06	0.82
2	1,3-Dimethylurate	0.82	0.89	1.09
3	1-Methylnicotinamide	1.53	1.45	0.94
4	2-Aminoadipate	1.05	0.87	0.83
5	2-Hydroxy-3-methylvalerate	1.18	1	0.84
6	2-Hydroxybutyrate	0.92	0.78	0.84
7	2-Hydroxyisobutyrate	0.99	1.03	1.03
8	2-Hydroxyphenylacetate	1.13	0.99	0.88
9	2-Octenoate	1.38	1.03	0.74
10	2-Oxoglutarate	0.55	0.68	1.22
11	2-Oxoisocaproate	1.04	0.85	0.81
12	2-Phenylpropionate	1.07	0.94	0.87
13	3-Hydroxybutyrate	0.92	1	1.09
14	3-Hydroxyisovalerate	1.14	1.07	0.93
15	3-Indoxylsulfate	1.01	0.84	0.82
16	3-Methyl-2-oxovalerate	1.24	1.08	0.87
17	3-Phenyllactate	1.22	1.19	0.97
18	4-Aminobutyrate	1.15	0.97	0.84
19	Acetate	1.3	1.37	1.05
20	Acetoacetate	0.92	0.8	0.87
21	Acetone	0.85	0.64	0.75
22	Adenine	0.56	5.59 *	9.9 *
23	Alanine	1.36 *	0.97	0.71 *
24	Anserine	1.32	1.15	0.87
25	Arginine	1.05	0.82	0.77
26	Asparagine	0.99	0.86	0.87
27	Aspartate	0.92	0.7	0.76
28	Betaine	1.28	1.2	0.94
29	Caffeine	1.25	0.9	0.71
30	Carnitine	1.06	0.84	0.79
31	Choline	0.97	0.88	0.9
32	Citrate	1.07	0.78	0.73 *
33	Creatine	2.81	10.61 *	3.77 *
34	Creatine phosphate	1.76 *	1.03	0.58 *
35	Dimethyl sulfone	1.64	1.46	0.89
36	Dimethylamine	0.71	0.77	1.08
37	Ethanolamine	1.08	1.12	1.04
38	Ethylene glycol	1	0.96	0.96
39	Formate	2.05 *	1.18	0.57 *
40	Galactarate	1.09	1.05	0.95
41	Galactose	0.94	1.27	1.34
42	Glucose	1.02	1.13	1.1
43	Glutamate	1.09	0.91	0.83
44	Glutamine	1.19 *	0.96	0.81 *
45	Glutarate	1.26	1.01	0.8
46	Glutathione	1.14	0.95	0.83
47	Glycerol	1.1	0.98	0.89
48	Glycine	1.35 *	0.94	0.69 *
49	Glycolate	1.38	0.85	0.61
50	Hippurate	1.28	1.52	1.18
51	Histamine	1.4	0.88	0.63
52	Histidine	0.92	0.85	0.92
53	Isobutyrate	1.39	1.36	0.97
54	Lactate	0.72	3.13	4.31
55	Lactose	1.58	1.78	1.12
56	Leucine	0.99	0.96	0.96
57	Malonate	1.29 *	0.98	0.76 *
58	Maltose	1.42	1.12	0.78
59	Mannitol	0.36 *	0.43 *	1.18
60	Methionine	1.22	0.93	0.76
61	Methylamine	1.08	0.83	0.77
62	Methylguanidine	0.81	1.04	1.28
63	Methylmalonate	0.89	0.74	0.83
64	N,N-Dimethylglycine	1.58	1.07	0.67
65	N-Acetylglucosamine	0.8	1.07	1.34
66	N-Acetyltyrosine	1.07	0.79	0.73
67	N-Methylhydantoin	1.81	1.34	0.73
68	N-Nitrosodimethylamine	1.31	1.05	0.8
69	N-Phenylacetylglycine	1.06	0.95	0.89
70	O-Acetylcholine	0.88	1.01	1.13
71	Propylene glycol	2.1	0.86	0.4
72	Pyruvate	1	0.94	0.93
73	Succinate	1.81	1.72	0.95
74	Succinylacetone	1.02	0.91	0.89
75	Taurine	0.76	1.63 *	2.12 *
76	Thymol	1.12	0.87	0.78
77	Trigonelline	2.05	1.06	0.51
78	Trimethylamine	1.5	1.03	0.68
79	Trimethylamine N-oxide	0.31 *	0.31 *	1
80	Urea	1.59 *	1.48 *	0.93
81	Uridine	1.56	0.9	0.57
82	Valine	1.13	1.02	0.9
83	cis-Aconitate	1.18	1.19	1
84	trans-Aconitate	0.78	0.85	1.09

* Metabolites showing significant differences according to Tukey’s multiple comparison test (*p* < 0.05).

## Data Availability

The original contributions presented in the study are included in the article; further inquiries can be directed to the corresponding authors.

## Abbreviations

ACL	Anterior cruciate ligament
ATP	Adenosine triphosphate
ANOVA	Analysis of variance
ARG1	Arginase-1
AGAT	Arginine:glycine amidinotransferase
BMI	Body mass index
DSS	Sodium trimethylsilylpropanesulfonate
EIMD	Exercise-induced muscle damage
FIFA	Fédération Internationale de Football Association
GPS	Global Positioning System
KWFF	Korea Women’s Football Federation
NMR	Nuclear magnetic resonance
(O)PLS-DA	(Orthogonal) partial least squares-discriminant analysis
PCA	Principal component analysis
PCr	Creatine phosphate
ROS	Reactive oxygen species
SD	Standard deviation
TCA	Tricarboxylic acid
TMAO	Trimethylamine N-oxide
VIP	Variable importance in projection
WK League	Women’s Korea League
WTS	Winter training season
